# Curriculum Innovation: Integrating Case-Based Learning With Seminar and Journal Club to Enhance Critical Thinking Skills in Residency Program

**DOI:** 10.1212/NE9.0000000000200213

**Published:** 2025-04-09

**Authors:** Sulena Sulena, Anil Kapoor, Ashwin Kulkarni, Ravinder Garg, Khushboo Bhagat, Akanksha Nagar, Kailash Charokar

**Affiliations:** 1 Division of Neurology, Guru Gobind Singh Medical College and Hospital, Faridkot, Punjab, India;; 2 Baba Farid University of Health Sciences, Faridkot, Punjab, India;; 3 Department of Medicine, People's College of Medical Sciences & Research Centre, Bhopal, Madhya Pradesh, India;; 4 Department of Medicine, Ramaiah Medical College, Banglore, Karnataka, India;; 5 Department of Medicine, Guru Gobind Singh Medical College, Faridkot, Punjab, India;; 6 Division of Neurology, Guru Gobind Singh Medical College, Faridkot, Punjab, India; and; 7 Department of Surgery, People's College of Medical Sciences & Research Centre, Bhopal, Madhya Pradesh, India.

## Abstract

**Background and Objectives:**

Effective management of complex neurologic cases requires strong critical thinking and clinical reasoning skills. However, traditional postgraduate education often lacks the structure to develop these competencies. We developed an integrated case-based learning (I-CBL) curriculum to enhance critical thinking skills of postgraduate internal medicine residents in neurologic patient assessments, evaluate its effectiveness as perceived by faculty, and improve residents' acceptance and satisfaction with its implementation.

**Methods and Curriculum Description:**

A prospective mixed-method (pretest and post-test) study with concurrent triangulation design was conducted among postgraduate internal medicine residents (residents) and faculty in a tertiary-care teaching hospital in India. The I-CBL approach was developed using the Theory of Change framework. I-CBL featured systematically planned seminars, journal presentations, and an in-depth case-based presentation tailored to different postgraduate year levels on the same topic within a month. Assessments included pretests and post-tests, Likert scale surveys, the Holistic Critical Thinking Scoring Rubric, and concept map evaluation. Knowledge acquisition and retention were measured immediately before, after, and again after 6 months of completing the topics. Quantitative data were analyzed using mixed-design analysis of variance while qualitative data underwent thematic analysis.

**Results and Assessment Data:**

All 30 residents (13 women, 17 men) and 10 faculty members participated. Residents showed significant knowledge gains from pretest to post-test, with retention maintained at 6 months (*p* < 0.05). Critical thinking skills improved notably in seminars (75%), case presentations (40%), and concept mapping (40%). Satisfaction was high, with 96% of residents and 92% of faculty reporting positive experiences. Residents appreciated the I-CBL approach for enhancing engagement, critical thinking, and clinical reasoning. Faculty recognized I-CBL's effectiveness in improving problem solving but cited challenges related to time constraints, workload, and participation equity.

**Discussion and Lessons Learned:**

The I-CBL curriculum effectively bridged theoretical knowledge and clinical practice, enhancing critical thinking, diagnostic skills, and teamwork. While satisfaction levels were high, challenges such as time constraints and faculty workload emerged. Proposed solutions include incorporating diverse cases, improving facilitation skills, and developing objective assessment tools. I-CBL can transform postgraduate neurology education by fostering critical thinking and clinical competence. Addressing implementation challenges can enhance its effectiveness, producing reflective, competent practitioners capable of managing complex neurologic cases and making confident, evidence-based decisions while navigating diagnostic dilemmas.

## Introduction and Problem Statement

While the field of neurology advances and continues to push the boundaries of innovation, passing on the legacy of practice to future physicians is paramount. Current medical education leaves significant gaps, particularly in developing critical thinking and clinical reasoning skills.^1^ Inadequacy and deficiency in core critical thinking and clinical reasoning skills, coupled with the arduous localization process in clinical neurology, often translate to poor patient outcomes, including suboptimal care and delayed diagnoses.^2,3^

Although related, critical thinking and clinical reasoning are distinct cognitive processes. Critical thinking involves evaluating, questioning, and reflecting on information, whereas clinical reasoning applies medical knowledge to clinical decision making.^4^ Critical thinking fosters the development of metacognitive skills, employing residents to assess their reasoning process and derive an integration of anatomy, physiology, and clinical acumen, which is the core of neurology.^5^ Residents use critical thinking to analyze differential diagnoses, evaluate evidence, and weigh treatment options. This is essential for sound clinical decision making, especially in neurology, where patients often present with ambiguous and multifaceted symptoms.^2,5^ Studies emphasize that critical thinking is not an innate skill but one that requires structured teaching interventions.^6^

Traditional didactic methods such as lectures fail to impart critical thinking skills.^7^ Therefore, active learning strategies such as group debates, case-based discussions, and problem-solving activities are implicated, empowering residents to question assumptions and manage uncertainty.^8^ Case-based learning (CBL) enhances critical thinking, boosts students' confidence in problem solving, and fosters the belief that it improves their academic performance and critical thinking skills.^9,10^ It is a powerful pedagogical approach using real-life clinical vignettes to develop critical thinking through diagnostic reasoning, clinical judgment, and problem-solving skills, creating confidence among residents to tackle neurologic cases independently, resulting in improved patient care and satisfaction.^11-13^

The demand for neurologists is rising in low-income and middle-income countries such as India, underscoring the urgent need to train competent health care professionals.^14^ Despite producing thousands of residents annually, postgraduate training still lacks sufficient emphasis on developing critical thinking skills.^15-17^ While competency-based medical education highlights the importance of critical thinking and clinical reasoning, traditional methods such as journal clubs and seminars remain underused for fostering collaborative, CBL.^18^ Innovative pedagogical approaches are essential. Medical education experts have also highlighted the need for uniform delivery standards, integrated evaluations, and mandatory inclusion of such training in residency curricula.^19^

This study introduces an innovative integrated CBL (I-CBL) approach that combines CBL with seminars and journal clubs to improve postgraduate internal medicine residents' (residents) understanding of complex neurologic cases. This initiative, extending beyond the standard residency curriculum, bridges the gap between theoretical knowledge and practical application. It also promotes collaborative learning, allowing residents to engage with cases and refine their clinical acumen actively.

The development of the I-CBL approach was guided by the Theory of Change (ToC) framework, which defined key processes, interventions, and change pathways.^20^ The ToC hypothesized that tailored, progressively challenging tasks would foster incremental skill improvement, supported by faculty mentorship and feedback. The curriculum was dynamic, incorporating real-time evaluations and feedback to ensure alignment with goals. This framework aligned with high-level evaluations from the Kirkpatrick model, assessing immediate learning outcomes and applying these skills in clinical practice.^21^ Embedding critical thinking in the curriculum equips future health care professionals to navigate diagnostic dilemmas and make evidence-based decisions confidently and competently.

## Objectives

We developed and introduced an I-CBL approach into the postgraduate internal medicine residency program with the following objectives:

1. Improve residents' knowledge and critical thinking skills in neurologic patient assessments.
2. Enhance faculty perceptions of I-CBL to improve critical thinking in neurologic patient evaluations.
3. Increase resident acceptance and satisfaction with I-CBL in developing critical thinking skills.

## Methods and Curriculum Description

This prospective mixed-method (pretest and post-test) study, with a concurrent triangulation design, was conducted among postgraduate internal medicine residents and faculty at a tertiary-care teaching hospital in India from September 1, 2023, to August 1, 2024.

### Participants and Training Context

The study involved 30 postgraduate internal medicine residents (residents) enrolled in a structured 3-year internal medicine residency program with rotations in neurology. Ten residents from each postgraduate year (PGY-1, PGY-2, and PGY-3) participated. Ten faculty members from the Department of Medicine participated as facilitators. All the residents and faculty were recruited using a purposive convenience sampling approach. Residents in India are clinical residents who have completed their undergraduate education and are involved in patient care while balancing theoretical learning and clinical practice. As they progress, residents take on greater patient management and decision-making responsibilities. This curriculum, designed to complement residents' academic, clinical, and research responsibilities, can be adapted for India's Doctorate of Medicine neurology training program, which follows the completion of an internal medicine residency.

### Curriculum Structure and Content

A core committee of experts meticulously developed an I-CBL curriculum using the ToC framework to enhance critical thinking among residents systematically. The ToC framework helped structure key processes and interventions to develop analytical, evaluative, and problem-solving skills in clinical settings. Expert faculty, curated educational materials, and robust institutional support formed the foundation for implementing targeted interventions.

The I-CBL approach featured systematically planned seminars, journal presentations, and an in-depth case-based presentation on the same topic within a month. This initiative, conducted outside the standard residency curriculum, provided scaffolding for deeper topic comprehension and connected cognitive and psychomotor domains with the latest advances.

**Figure 1:**
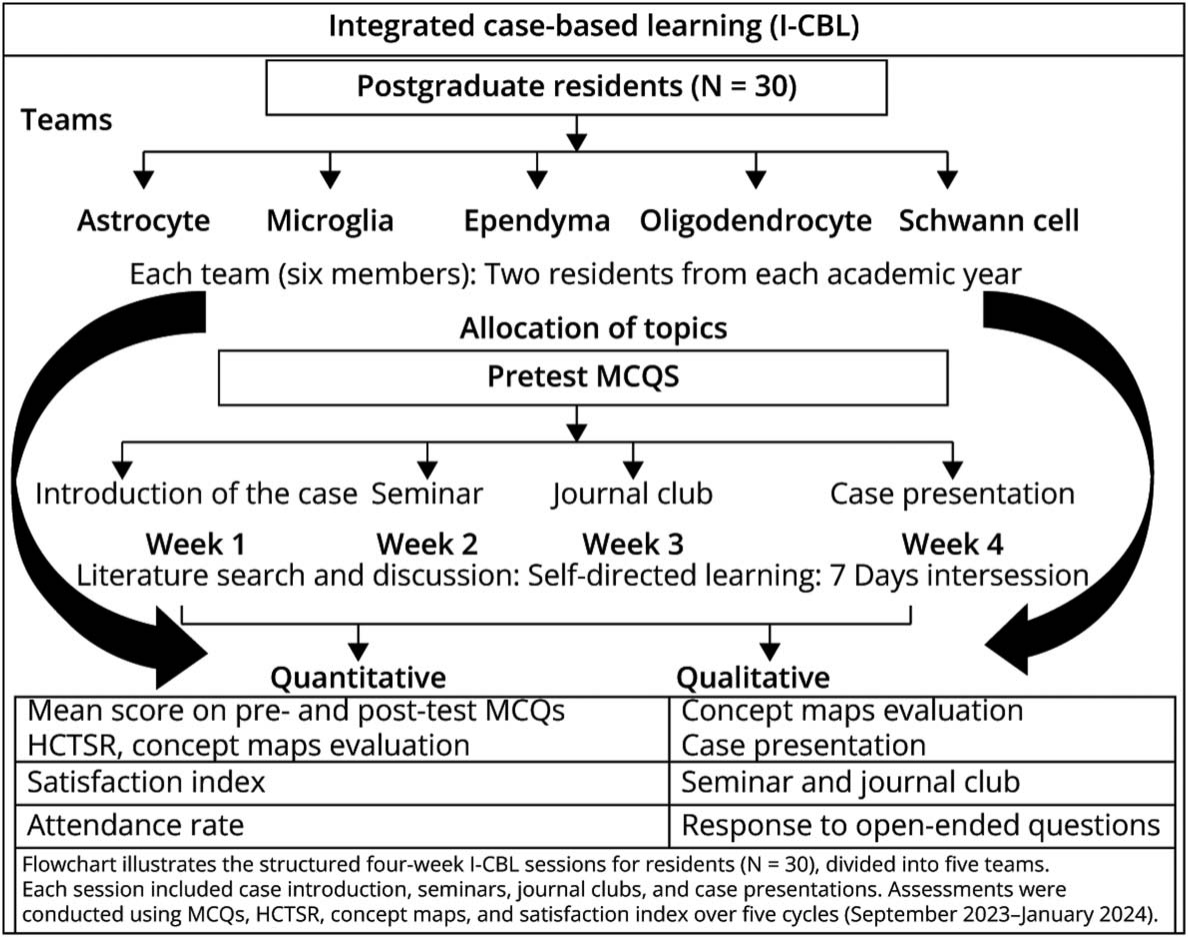
Flowchart Depicting the I-CBL Curriculum Methodology HCTSR = Holistic Critical Thinking Scoring Rubric; I-CBL = integrated case-based learning; MCQ = multiple-choice question.

Residents were divided into 5 teams, each with 6 members from different PGY levels (Figure 1). This structure promoted collaborative learning by offering diverse perspectives and fostering peer learning. Each postgraduate year had tailored activities: PGY-1 residents attended seminars to introduce foundational knowledge and promote critical discussion; PGY-2 residents participated in journal clubs to critically appraise research evidence and strengthen evidence-based reasoning; PGY-3 residents presented complex clinical cases to refine diagnostic reasoning and decision making. In addition, concept mapping was used to consolidate knowledge and link theory with practice visually. This structured progression enabled residents to develop skills in analyzing, evaluating, and synthesizing clinical information. Faculty mentorship and standardized assessments, including the Holistic Critical Thinking Scoring Rubric (HCTSR),^22^ ensured consistent evaluation and improvement. Real-time feedback helped participants refine their reasoning abilities.

### Curriculum Implementation

Before the intervention, dedicated sensitization sessions were conducted for faculty members and residents to clarify roles and expectations. Faculty sessions emphasized methodologies for conducting I-CBL sessions, standardizing assessments, and providing effective guidance. Resident sessions highlighted the program's objectives, structure, and expectations, creating a shared understanding among participants. The curriculum was implemented over 5 months (September 2023–January 2024). Each monthly session focused on a specific neurology topic related to the approach to patients with stroke, spinal cord disorders, movement disorders, peripheral neuropathy, and altered sensorium.

### Curriculum Assessment

The effectiveness of the curriculum was evaluated using a framework aligned with the study objectives. Residents' knowledge and critical thinking were assessed through preintervention and postintervention multiple-choice questions (MCQs), the HCTSR, and concept map evaluations. Faculty and residents' perceptions and satisfaction were gathered through Likert scale surveys and open-ended feedback. Qualitative data from these responses were analyzed to explore their perceptions of the I-CBL approach. Six months later, a follow-up survey was conducted to evaluate the long-term impact.

#### Tool Development and Validation

The core committee developed assessment tools emphasizing face validity, content validity, and alignment with the program objectives. Two Likert scale surveys—1 for residents (12 items) and 1 for faculty (8 items)—were used to evaluate the CBL curriculum's acceptability and effectiveness. Responses ranged from “strongly disagree” (1) to “strongly agree” (5), with open-ended questions providing qualitative feedback. The surveys demonstrated strong reliability, with Cronbach α values of 0.93 (faculty) and 0.83 (residents). Satisfaction levels were scored on a 9-point scale (low: 1–3, medium: 4–6, high: 7–9) as an additional measure of success. A follow-up survey 6 months later assessed long-term impacts, including perceived usefulness, confidence levels, and knowledge retention.

#### Knowledge Acquisition and Retention

Knowledge acquisition was measured through MCQs administered before and after the intervention, focusing on diagnostic reasoning and evidence-based decision making. The MCQs were validated for clarity and relevance. Six months later, the same MCQs were used to assess retention, with questions reshuffled to minimize recall bias. Consistent quality control ensured reliable assessments.

#### Holistic Critical Thinking Scoring Rubric

The HCTSR^22^ evaluated critical thinking across 3 activities: seminar presentations, journal clubs, and case presentations. Residents were assessed on their ability to explain neurologic concepts, critically analyze research, and apply evidence-based reasoning. Faculty underwent training sessions to ensure consistent and accurate rubric application, supported by calibration workshops. The inter-rater reliability was high (83%). The scoring criteria included clarity (the ability to communicate reasoning effectively), relevance (connecting discussions to the case or problem), depth (thorough exploration of the issue from multiple perspectives), accuracy (application of valid information and logical reasoning), and fairness (avoiding bias and incorporating balanced viewpoints).

#### Concept Map Assessment

Concept maps were evaluated using a rubric that addressed breadth, interconnectivity, descriptive links, link efficiency, and layout.^23^ Residents' synthesis and application of knowledge were reflected in their ratings of the maps, which were rated as unacceptable, acceptable, good, or exemplary.

Two faculty members independently reviewed each session and map, resolving discrepancies through consensus.

### Statistical Analysis

#### Quantitative Data

Data were entered into Microsoft Excel and analyzed using SPSS version 20.0.0 software. Descriptive statistics, including means and standard deviations, were calculated to summarize the data. Normality was assessed using the Shapiro-Wilk test. Pretest, post-test, and 6-month retention data were analyzed using mixed-design analysis of variance (ANOVA) to evaluate the effects of the educational intervention on knowledge acquisition across neurologic topics. Repeated-measures ANOVA was used to assess differences in test scores at the 3 time points for each topic. Partial eta squared was calculated to determine the effect size of the intervention. A *p* value of <0.05 was considered statistically significant. The percentage of residents and faculty selecting each response option was calculated for feedback analysis. The satisfaction index was determined by averaging responses on a 5-point Likert scale (strongly agree = 5, agree = 4, neutral = 3, disagree = 2, strongly disagree = 1).

Faculty used the HCTSR criteria to assess participants, with ratings assigned on a 4-level scale: weak (1), unacceptable (2), acceptable (3), and strong (4). Progress was evaluated by analyzing trends in ratings over the 5-month intervention. Each rating was numerically assigned (1–4), and improvement was calculated as the percentage change between the first and last month's scores using the following formula: Improvement (%) = ([Score in the fifth month − Score in the first month]/4) × 100. A similar approach was used to assess concept maps using the rubric.

#### Qualitative Data

Narrative data from open-ended questions were analyzed using thematic analysis with inductive coding. Responses were transcribed, coded, categorized into themes, and reviewed iteratively for consistency to ensure reflexivity and minimize bias. Two independent researchers conducted peer-to-peer debriefing. Data analysis was anonymous, and participatory validation was incorporated where feasible.

### Standard Protocol Approvals, Registrations, and Participant Consents

The institutional ethics committee granted ethical approval (GGS/IEC/04, dated August 24, 2023), and informed written consent was obtained from all the participants.

### Data Availability

Anonymized data not published within this article will be available to the qualified investigator on reasonable request.

## Results and Assessment Data

### Participant Demographics

The study included 30 residents with a mean age of 26.6 ± 1.4 years (13 women, 43.3%; 17 men, 56.7%). Ten faculty members (F: M = 1:1) with a mean age of 36.4 ± 7.2 years and an average teaching experience of 6.4 ± 3.8 years served as facilitators for the I-CBL sessions.

### Knowledge Acquisition

The I-CBL sessions significantly enhanced residents' knowledge of neurologic topics. Improvements were evidenced by statistically significant increases in mean scores from the pretest to the post-test and at the 6-month follow-up (*p* < 0.05; Table 1). Large effect sizes were observed in most topics (Cohen *d* > 0.8), reflecting immediate and sustained knowledge retention.

**Table 1:** Mean Pretest, Post-test, and 6-Month Follow-Up Scores of MCQs on the Topics

Sections Approach to a patient with	No. of MCQs (marks)	Pretest Mean ± SD	Post-test Mean ± SD	Retention (6 mo) Mean ± SD	*F* value	*p* Value	η_p_^2^
1. Stroke	10 (26)	15.8 ± 5.7	19.7 ± 3.8	21.6 ± 2.7	21.4	0.000	0.50
2. Spinal cord	15 (15)	8.6 ± 1.7	10.5 ± 1.9	11.8 ± 1.4	58.1	0.000	0.81
3. Movement disorders	10 (10)	5.70 ± 2.0	7.6 ± 1.4	8.7 ± 0.9	38.3	0.000	0.97
4. Peripheral neuropathy	5 (5)	1.7 ± 0.7	3.0 ± 0.91	3.5 ± 0.7	53.6	0.000	0.97
5. Altered sensorium	10 (22)	11.6 ± 3.3	14.3 ± 2.8	16.5 ± 3.0	25.0	0.000	0.97

Abbreviation: MCQ = multiple-choice question.

### Development of Critical Thinking Skills

**Figure 2:**
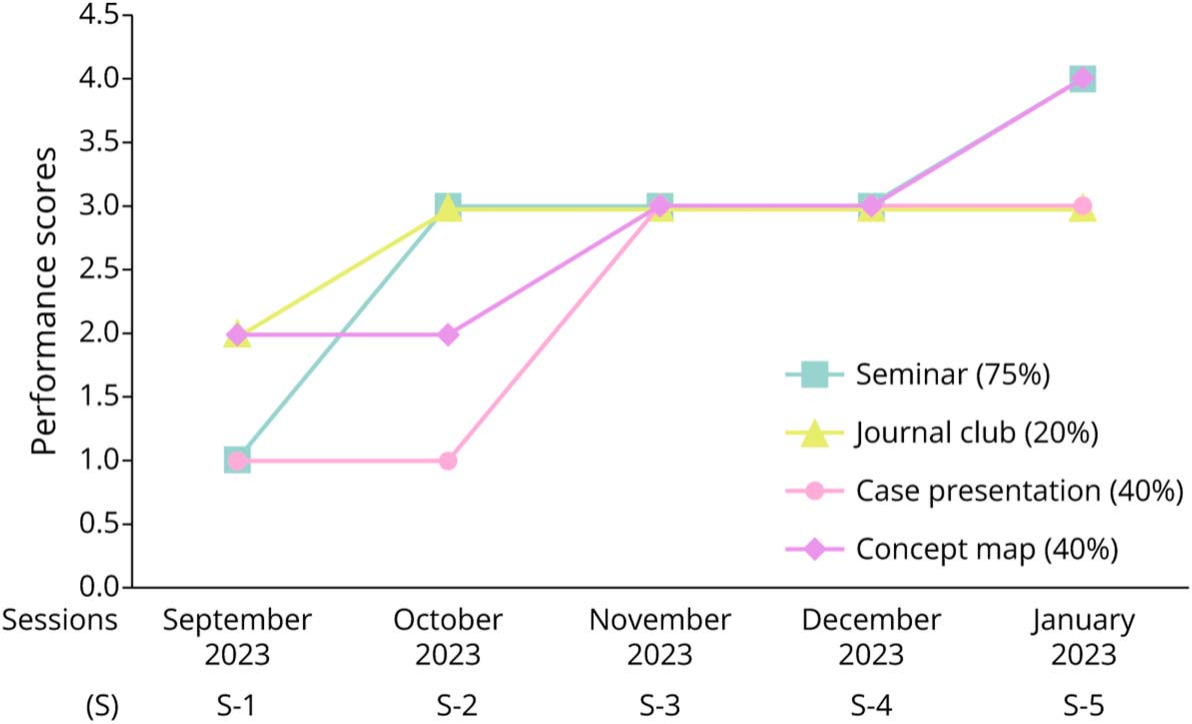
Progressive Evaluation of Residents' Performance Across Monthly I-CBL Sessions I-CBL = integrated case-based learning.

Faculty evaluations using the HCTSR indicated marked improvements in residents' critical thinking skills over the 5-month intervention (Figure 2). Seminars showed a 75% increase in scores, evolving from weak to strong performance, with improved clarity, depth, and analysis rigor. Case presentations demonstrated a 40% improvement, highlighting progress in diagnostic reasoning and reflective thinking by the third session. Journal club sessions improved by 20%, transitioning from unacceptable to acceptable, although scores plateaued in later sessions. Concept mapping activities improved by 40%, indicating an enhanced ability to synthesize and visually organize complex information.

### Satisfaction Levels

The mean satisfaction index among residents was 96%, with 78% (N = 23) reporting high and 22% (N = 7) reporting medium satisfaction. All participants agreed that CBL enhanced their critical thinking, diagnostic reasoning, and teamwork skills (Table 2). Faculty reported a mean satisfaction index of 92%, with 30% (N = 3) expressing high satisfaction and 70% (N = 7) reporting medium satisfcation. All faculty members acknowledged I-CBL's effectiveness in fostering critical thinking and problem solving.

**Table 2:** Perception of Residents Toward I-CBL

S. no.	Questions	Disagree N (%)	Neutral N (%)	Agree N (%)
1	I-CBL helped improved my critical analysis skills	0 (0)	1 (3)	29 (97)
2	I-CBL improved my problem-solving skills	0 (0)	2 (7)	28 (93)
3	I-CBL improved my clinical reasoning skills	0 (0)	1 (3)	29 (97)
4	I-CBL method of teaching-learning was interesting	0 (0)	2 (7)	28 (93)
5	I-CBL provided opportunities for interaction and collaboration and team work with other students	0 (0)	0 (0)	30 (100)
6	I-CBL motivated for self-directed learning	1 (3)	1 (3)	28 (93)
7	I-CBL improved my diagnostic approach to a patient with neurologic disease	0 (0)	0 (0)	30 (100)
8	I feel more confident in case presentation after using I-CBL	0 (0)	0 (0)	30 (100)
9	I-CBL provided me experiential learning	0 (0)	1 (3)	29 (97)
10	I-CBL helped develop high order knowledge among students	0 (0)	0 (0)	30 (100)
11	I am satisfied with use of I-CBL as a teaching learning method	2 (7)	0 (0)	28 (93)
12	I am satisfied that I-CBL helped improve critical analysis skills	1 (3)	0 (0)	29 (97)
Mean satisfaction index		97%		

Abbreviation: I-CBL = integrated case-based learning.

### Qualitative Insights

**Figure 3:**
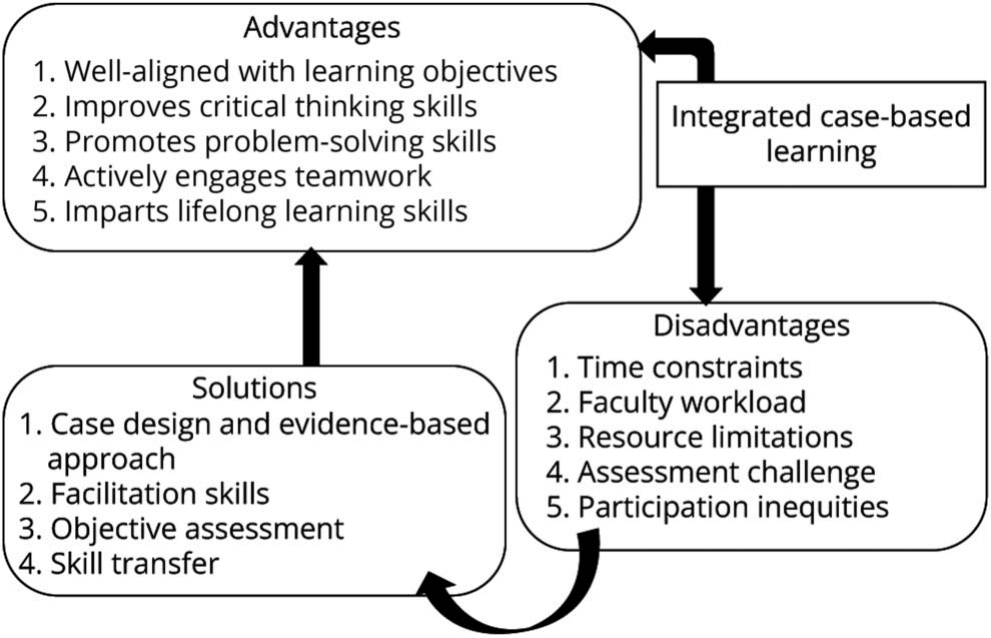
Perception of Faculty Toward I-CBL I-CBL = integrated case-based learning.

The analysis of open-ended responses revealed 2 key themes for residents and 3 for faculty perceptions (Figure 3). Each theme is detailed as follows, with representative quotes illustrating their responses.

### Residents' Responses

#### Theme 1: Positive Perceptions

##### Subtheme 1: Enhanced Engagement

Residents found I-CBL far more interactive and engaging. The dynamic structure of I-CBL sessions required active participation, discussion, and direct application of theoretical knowledge to real-world scenarios. This interactive environment motivated residents and fostered a sense of ownership over their learning process. I-CBL encouraged them to explore topics in depth, ask questions, and seek out information independently. One resident said, “The sessions motivated me to learn self-directedly and were highly engaging.”

##### Subtheme 2: Improved Critical Thinking

I-CBL challenged residents to critically assess clinical evidence, weigh different diagnostic possibilities, and justify their decisions. By exposing them to varied case scenarios and requiring them to defend their reasoning, I-CBL honed their analytical and decision-making skills. Residents appreciated how the sessions pushed them to consider multiple perspectives and think deeply about diagnostic processes, enhancing their ability to solve complex neurologic problems. One of the quotes is, “I-CBL is a priceless tool for sharpening analytical skills and enhanced my ability to evaluate… and solve diagnostic problems in neurology.”

##### Subtheme 3: Development of Clinical Reasoning

Residents reported that I-CBL facilitated the development of clinical reasoning and diagnostic proficiency through exposure to realistic case scenarios. They learned to approach cases systematically and solve problems within time constraints. This method enhanced their understanding of neurologic concepts and boosted their confidence in handling intricate patient cases. The real-world relevance of the cases made the learning process more meaningful and practical.

##### Subtheme 4: Collaboration and Communication

I-CBL's group activities improved teamwork and the ability to articulate complex ideas, mirroring health care settings. Residents worked in teams to solve cases, discuss findings, and present their conclusions. This collaborative approach improved their ability to articulate complex ideas clearly, negotiate differing viewpoints, and learn from peers. The teamwork aspect helped build essential communication skills vital in multidisciplinary health care settings. Residents quote, “Working in teams and presenting the tasks collaboratively enhanced my learning experience.”

#### Theme 2: Negative Perceptions

##### Subtheme 1: Time Commitment

Residents cited the substantial time required for I-CBL sessions as a major challenge. The preparation, which included researching cases, understanding the background, and participating in discussions, was demanding and often conflicted with their other academic and clinical responsibilities. Residents quote, “I appreciate the value of I-CBL, but the time commitment is just too much. I barely have time to keep up with my clinical and academic work.”

##### Subtheme 2: Individual Learning Styles

While I-CBL's collaborative approach benefited many, it did not align with everyone's preferred learning style. Some residents found the group-based nature of I-CBL less effective, as they preferred more structured, individual learning environments. Residents quote, “I-CBL doesn't match my learning style. I find it more effective to study independently and then clarify my doubts later.”

### Faculty Responses

#### Theme 1: I-CBL's Impact on Critical Thinking and Problem Solving

Faculty members unanimously agreed that I-CBL effectively improved residents' critical thinking and problem-solving skills, particularly in evaluating complex neurologic cases. They viewed CBL as well aligned with the learning objectives and the practical needs of residents, fostering an environment of active engagement, collaboration, and lifelong learning. A faculty member noted, “I-CBL encourages residents to take ownership of their learning, actively seeking information and exploring different perspectives, promoting lifelong learning habits.”

#### Theme 2: Challenges in Implementing I-CBL

Faculty acknowledged several challenges in implementing I-CBL, including time constraints that made it difficult to balance in-depth discussions with covering the full syllabus. They also identified the increased workload, the lack of materials and institutional support, and the challenge of measuring critical thinking beyond subjective observations. Faculty also noted participation inequalities, with quieter residents sometimes being overshadowed. One faculty member explained, “Ensuring all residents participate equally can take time and effort. Sometimes, the quieter ones get left out.”

#### Theme 3: Solutions to Challenges

To address the challenges, faculty members suggested stimulating diagnostic reasoning with diverse and more challenging cases, such as atypical neurologic presentations. They also recommended providing more resources, improving facilitation skills, and developing objective tools to measure critical thinking and ensure skill transfer. One faculty member stated, “Including more diverse and challenging cases, like atypical presentations, would be great for pushing diagnostic reasoning.”

### Follow-Up Findings

A follow-up survey revealed that most residents valued the integration of I-CBL. They reported increased confidence in managing neurologic cases and a strong preference for continuing to implement I-CBL with more frequent sessions (Tables 3 and 4).

**Table 3:** Six-Month Follow-Up: A Resident Survey on I-CBL

	Question	Response categories	Frequency (N), percentage (%)
1	Was “integration of CBL with seminar and journal club” helpful	Very helpful	28 (93)
		Slightly helpful	2 (7)
2	Most useful part of the experience	Case-based learning	21 (70)
		Seminars	21 (70)
		Journal clubs	23 (77)
		Integration of CBL with seminar and journal club	24 (80)
3	Should periodic integration of I-CBL continue?	Definitely yes	25 (83)
		Probably yes	5 (17)
4	Preferred frequency for I-CBL Integration?	More than once a month	15 (50)
		Once a month	13 (43)
		Every few months or once a year	2 (7)

Abbreviation: I-CBL = integrated case-based learning.

**Table 4:** Follow-Up Survey 6 Months After I-CBL: Comparison of Residents' Preintervention and Postintervention Experience Completing the Tasks

Questions	Preintervention Mean ± SD	Postintervention Mean ± SD	*T* value	*p* Value
Q5.1 Elicit a comprehensive patient history from neurology patients	4.5 ± 3.6	8.2 ± 1.7	6.9	0.001
Q5.2 Localize a lesion using data gathered from the history and neurologic exam	4.5 ± 3.6	7.9 ± 2.4	5.8	0.001
Q5.3 Perform the neurologic examination of the cases	4.5 ± 3.6	8.1 ± 2.2	6.9	0.001
Q5.4 Perform the critical analysis of the neurologic cases	4.4 ± 3.5	7.9 ± 2.5	6.2	0.001
Q5.5 Plan appropriate diagnostic tests for neurology patients	4.4 ± 3.6	8.2 ± 1.7	6.9	0.001

Abbreviation: I-CBL = integrated case-based learning.

## Discussion and Lessons Learned

CBL is a student-centered pedagogy that uses real clinical cases to bridge the gap between theoretical knowledge and real-world practice through inquiry-based methods.^24^ This study introduced an innovative I-CBL as a teaching method for a postgraduate residency program in internal medicine, specifically focusing on neurologic disorders. Our findings demonstrate both short-term and long-term effectiveness in enhancing residents' critical thinking, problem-solving abilities, and knowledge acquisition. High satisfaction rates among residents (96%) and faculty (92%) reflect positive outcomes in Kirkpatrick Level 1 evaluation.

Traditional teaching methods have their merits but may not fully prepare residents for the complexities of critical analysis and clinical reasoning in neurology. CBL offers a more comprehensive approach by allowing residents to apply their knowledge to real-world clinical scenarios.^24^ Seminars generate guided interactions among students on a theme, developing cognitive abilities such as applying concepts and principles.^25^ Journal club presentations critically assess recent advances in the literature, helping participants develop cognitive skills and stay up-to-date with the latest research.^26,27^ Residents can gain extra advantages of each approach by integrating CBL using real patients with seminars and journal club presentations. This multifaceted approach can better prepare students for the complexities of neurologic cases by combining the benefits of active and collaborative learning, applying knowledge to diagnose and manage real patients, engaging in guided discussions, critically analyzing research, and staying current with the field.

While CBL has been extensively studied, there is a dearth of research on its integrated application in postgraduate medical education.^26,28^ Significant improvements in knowledge assessment scores across various neurologic topics were observed after sessions (*p* < 0.05; Table 1), aligning with Kirkpatrick Level 2 evaluation of learning outcomes. These results are consistent with a recent meta-analysis indicating enhanced academic performance and active critical analysis of cases (*p* < 0.001).^29^ Compared with other teaching methods, CBL yields superior results in short-answer and matching questions, likely because of the provision of resources and background information that guided the learning process and reduced student burden.^30^

The HCTSR and the rubric used for concept maps revealed progressive improvement in residents' critical analysis and evaluation skills during seminars, journal clubs, and case presentations, aligning with Kirkpatrick Level 3 evaluation (behavior) (Table 2). These results support the effectiveness of I-CBL in fostering critical thinking skills and align with previous research demonstrating the HCTSR's value in medical education.^31^ The high inter-rater reliability of HCTSR evaluations (83%) underscores the consistency of the assessment process. While the intervention significantly enhanced critical thinking in specific domains, the plateau in journal clubs and case presentations suggests potential areas for refinement. These findings indicate that the structured I-CBL approach positively influenced critical thinking in this learner population. However, further analysis and targeted modifications are needed to sustain and amplify its impact.

I-CBL effectively fostered critical thinking skills by involving residents in real-life situations and promoting active participation. Residents and faculty reported high satisfaction with the I-CBL, with a 96% and 92% satisfaction index, respectively. All residents appreciated the engaging and interactive nature of I-CBL, which contributed to developing higher order knowledge, improved diagnostic approaches, and enhanced collaboration. These findings resonate with those of previous studies highlighting CBL's ability to promote enthusiasm, active learning, collaboration, and critical thinking skills.^24,32^ Only 1 resident (3.33%) expressed concerns about time constraints and potential mismatch with learning styles; these issues can be addressed through comprehensive implementation checklists.^33,34^

Faculty recognized I-CBL's ability to cultivate critical thinking and problem-solving skills. This aligns with a meta-analysis reporting that CBL promotes a dynamic learning environment conducive to clinical reasoning and decision making among medical and pharmacy students.^30,32^ CBL encourages self-directed learning and analysis, mirroring real-world clinical reasoning processes.^11,24^ Faculty also perceived I-CBL as well aligned with learning objectives and practical requirements, showcasing its potential in neurologic evaluations. Existing literature reveals that residents engaged in CBL performed better in clinical examinations, suggesting a positive transfer of acquired skills to clinical applicability.^33,34^ Thus, I-CBL offers a robust pedagogical tool for postgraduate neurologic education in India, demonstrating its alignment with the recent National Medical Commission's focus on competency-based medical education.^18^

A follow-up survey after 6 months revealed that residents found the I-CBL approach helpful and valuable, leading to increased confidence in performing and completing neurologic tasks and a strong desire for more frequent sessions (Table 4). These findings align with existing educational research, highlighting the effectiveness of structured interventions in enhancing residents learning outcomes.^35,36^

I-CBL fosters collaboration and teamwork, creating a dynamic learning environment. This aligns with research highlighting collaborative learning and active participation as key factors in knowledge retention.^37^ Designing engaging lessons and activities that use active learning methods is crucial for maximizing student immersion and long-term retention.^38^ This study highlights the short-term and long-term positive impact of I-CBL on residents' development. CBL encompasses crucial skills such as clinical reasoning, differential diagnosis, knowledge integration, and lifelong learning skills.^39,40^ It equips residents with the ability to adapt and evolve in the ever-changing field of medicine.^39^

Our faculty reported several implementation challenges, including time constraints, workload, resource limitations, assessment difficulties, and uneven student participation, which align with existing literature.^32,37,40^ To overcome these challenges, faculty recommended incorporating diverse cases, enhancing facilitation skills, using objective assessment tools, and promoting skill transfer, all of which are essential for effectively implementing I-CBL.

Despite the study's small sample size and single-center design, our findings suggest that I-CBL is an effective method for enhancing knowledge acquisition, critical thinking, and satisfaction among postgraduate residents and faculty. While the absence of a control group necessitates cautious interpretation, the significant improvements observed within the cohort highlight the intervention's potential value. Furthermore, multicenter studies are needed to explore the generalizability of the I-CBL model across diverse medical institutions.

In conclusion, integrating CBL with seminars and journal clubs enhances critical thinking and satisfaction among internal medicine residents and faculty in neurology education. This tailored I-CBL approach aligns with the maturity of postgraduate learners, optimizing outcomes by developing reflective practitioners who excel in managing complex neurologic cases and making confident, evidence-based decisions. Expanding the use of this innovative model could shape future postgraduate medical education, fostering excellence in clinical practice and critical decision making.

## Glossary

ANOVA: analysis of variance
HCTSR: Holistic Critical Thinking Scoring Rubric
CBL: case-based learning
I-CBL: integrated CBL
MCQ: multiple-choice question
PGY: postgraduate year
ToC: Theory of Change
